# Replication of Real-World Evidence in Oncology Using Electronic Health Record Data Extracted by Machine Learning

**DOI:** 10.3390/cancers15061853

**Published:** 2023-03-20

**Authors:** Corey M. Benedum, Arjun Sondhi, Erin Fidyk, Aaron B. Cohen, Sheila Nemeth, Blythe Adamson, Melissa Estévez, Selen Bozkurt

**Affiliations:** 1 Flatiron Health, Inc., 233 Spring Street, New York, NY 10003, USA; corey.benedum@flatiron.com (C.M.B.); arjun.sondhi@flatiron.com (A.S.); erin.fidyk@flatiron.com (E.F.); aaron.cohen@flatiron.com (A.B.C.); sheila.nemeth@flatiron.com (S.N.); badamson@flatiron.com (B.A.); selen.bozkurt@flatiron.com (S.B.); 2 Department of Medicine, NYU Grossman School of Medicine, New York, NY 10016, USA; 3 Comparative Health Outcomes, Policy and Economics (CHOICE) Institute, University of Washington, Seattle, WA 98195, USA

**Keywords:** electronic health records, machine learning, natural language processing, cancer, real-world data, artificial intelligence, quality, oncology, real-world evidence

## Abstract

**Simple Summary:**

Obtaining and structuring information about the characteristics, treatments, and outcomes of people living with cancer for research purposes is difficult and resource-intensive. Oftentimes, this information can only be found in electronic health records (EHRs). In response, researchers use natural language processing with machine learning (ML extraction) techniques to extract information at scale. This study evaluated the quality and fitness-for-use of EHR-derived oncology data curated using ML extraction, relative to the standard approach, abstraction by trained experts. Using patients with lung cancer from a real-world database, we performed replication analyses demonstrating common analyses conducted in observational research. Eligible patients were selected into biomarker- and treatment-defined cohorts, first with expert-abstracted then with ML-extracted data. The study’s results and conclusions were similar regardless of the data curation method used. These results demonstrate that high-performance ML-extracted variables trained on expert-abstracted data can achieve similar results as when using abstracted data, unlocking the ability to perform oncology research at scale.

**Abstract:**

Meaningful real-world evidence (RWE) generation requires unstructured data found in electronic health records (EHRs) which are often missing from administrative claims; however, obtaining relevant data from unstructured EHR sources is resource-intensive. In response, researchers are using natural language processing (NLP) with machine learning (ML) techniques (i.e., *ML extraction*) to extract real-world data (RWD) at scale. This study assessed the quality and fitness-for-use of EHR-derived oncology data curated using NLP with ML as compared to the reference standard of expert abstraction. Using a sample of 186,313 patients with lung cancer from a nationwide EHR-derived de-identified database, we performed a series of replication analyses demonstrating some common analyses conducted in retrospective observational research with complex EHR-derived data to generate evidence. Eligible patients were selected into biomarker- and treatment-defined cohorts, first with expert-abstracted then with ML-extracted data. We utilized the biomarker- and treatment-defined cohorts to perform analyses related to biomarker-associated survival and treatment comparative effectiveness, respectively. Across all analyses, the results differed by less than 8% between the data curation methods, and similar conclusions were reached. These results highlight that high-performance ML-extracted variables trained on expert-abstracted data can achieve similar results as when using abstracted data, unlocking the ability to perform oncology research at scale.

## 1. Introduction

**Figure 1:**
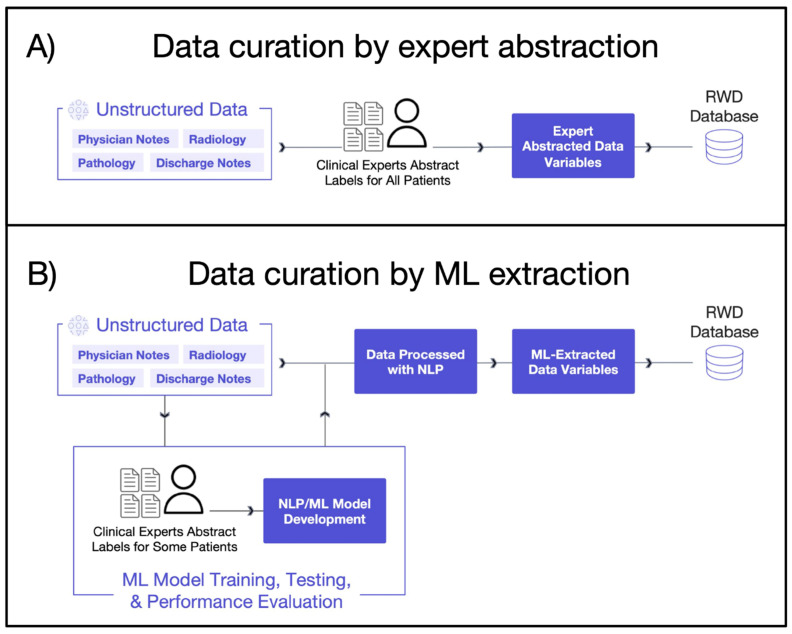
Conceptual diagram of EHR data curation highlighting approaches to define variables. Abbreviations: ML: machine learning; NLP: natural language processing; RWD: real-world data. (Panel (**A**)): Unstructured data are reviewed by trained clinical abstractors to collect relevant data from patients’ charts. (Panel (**B**)): Process for developing models and extracting information from unstructured data sources from the patient’s chart.

The digitization of healthcare, driven in part by the Health Information Technology for Economic and Clinical Health (HITECH) Act signed into US law in 2009, has increased the availability of real-world data (RWD). Likewise, the demand for real-world evidence (RWE) to support comparative effectiveness research and better understand patient populations and clinical outcomes has grown [1,2,3,4]. Despite this growth in available patient data, 80% of RWD is in unstructured free-text and requires manual curation and processing to be usable for analysis purposes [4,5]. Valuable information regarding the characteristics, treatments, and outcomes of people living with cancer is found in unstructured text documents stored within electronic health records (EHRs). How to access and analyze this information at scale for RWE generation is a massive challenge. The standard method of data curation through expert human abstraction is resource-intensive and time-consuming, limiting the number of patients available for research purposes [5,6,7]. In response, natural language processing (NLP) with machine learning (ML) techniques (i.e., *ML extraction*) is increasingly being applied to EHR data for more efficient and scalable generation of RWD (Box 1). ML extraction techniques can learn and recognize language patterns to provide automated solutions for extracting clinically relevant information, thereby enabling research and RWE generation at scale [8] (Figure 1). As researchers seek to understand smaller and more niche patient populations and stay on top of rapidly evolving standards of care, the need to generate RWD quickly and for more patients is becoming increasingly important. By automatically processing free text to extract clinical information, ML extraction can generate RWD at a speed and scale that far exceeds manual data curation and thereby meet the evolving needs of clinical and health outcomes research. For example, ML extraction can scan an enormous population, searching for rare patient characteristics buried in unstructured EHR data sources to select niche populations and unlock larger cohort sizes than would be feasible with expert abstraction.

Box 1 **Defining key terms.**

*Natural language processing* (NLP) is a tool used to enable computers to analyze, understand, derive meaning from, and make use of human language. Often, NLP is applied to identify and extract relevant information from unstructured data. The output of this document processing is a set of features which capture document structure, chronology, and key clinical terms or phrases. These features can then serve as the inputs for a machine learning model.

*Machine learning* (ML) can also be used to perform NLP to extract data from unstructured sources. ML models are designed to learn to perform tasks without being explicitly programmed to do so. For example, a ML model can be trained to learn what keywords or phrases found in a patient’s clinical documents are associated with a variable of interest.

Most regulatory guidance related to ML has primarily focused on evaluating ML models and software as a medical device [9]. Until recently, there had been limited regulatory guidance regarding the best practices for evaluating ML-extracted RWD, aside from an overarching agreement on the need for transparent methods and processes. Both the UK’s NICE RWE framework and FDA’s RWE guidance ultimately aim to deliver on this by improving RWE quality through detailed guidance on what constitutes RWD, data curation, and analysis reporting standards, measuring quality and addressing limitations such as missing data or information bias [10,11,12]. While there is growing attention and guidance around RWD at large, there remains a gap regarding the evaluation of the quality and performance of ML-extracted RWD.

In response to this gap, we previously developed a research-centric evaluation framework to evaluate ML-extracted RWD and provide insights on model performance, strengths and limitations, and fitness-for-use [6]. This framework primarily focuses on evaluating a single ML-extracted variable, independent of the output of other ML extraction models. Univariable analyses include characterizing the model’s overall performance and performance stratified by key patient characteristics, quantitative error analysis, where the characteristics of correctly and incorrectly extracted patients are compared, to understand the potential for systematic bias due to model errors, and finally a comparison of the outcomes between cohorts selected by the model as compared to expert abstraction. While understanding the quality of the extracted data for individual variables is important, univariable evaluations cannot describe how model errors may interact together and potentially introduce bias when multiple ML-extracted variables are used in combination for research purposes (e.g., selection bias due to poor model performance in select sub-groups or information bias, resulting in shifts in covariate distributions) [13,14]. As such, replication analyses leveraging datasets containing several ML-extracted variables are integral to understanding the reproducibility of analytic results and scientific conclusions when using data curated via ML extraction versus expert abstraction.

We identified common archetypes describing how EHR-derived data are used in observational research. These archetypes include but are not limited to: (1) defining baseline characteristics, (2) describing the natural history of disease, (3) balancing populations, and (4) measuring treatment comparative effectiveness. For this study, we designed example oncology retrospective studies for each archetype that require information from both unstructured and structured complex EHR data, and assessed whether the use of ML-extracted data leads to the same analytic conclusions when used in place of expert-abstracted data for each. The retrospective studies we designed include two study populations: a biomarker-defined cohort and a treatment-defined cohort. These populations were selected because the ability of ML-extracted data to select these cohorts unlocks the ability to perform comparative effectiveness research and understand outcomes, including in rare populations that can benefit from targeted therapy. For the biomarker-defined cohort, we selected patients with a ROS1 rearrangement (in addition to ROS1-negative patients) to evaluate the ability of ML-extracted data to select a cohort with a low prevalence. For the treatment-defined cohort, we chose patients who received first line (1L) treatment with either bevacizumab–carboplatin–paclitaxel (BCP) or carboplatin–paclitaxel (CP). Since the goal of this study is to compare ML extraction with expert abstraction and not to contribute to the scientific understanding of cancer treatment, we intentionally selected populations that are well established in the literature.

## 2. Materials and Methods

We developed a series of retrospective study replications in non-small cell lung cancer (NSCLC) to compare conclusions based on ML-extracted data relative to expert-abstracted data. Two research questions were defined to illustrate the common archetypes for RWD use cases:

1. *What is the relationship between a rare cancer biomarker alteration and patient survival?*
2. *What is the comparative effectiveness of two cancer treatment regimens?*

For each research question, we defined an analytic cohort and selected patients who met the cohort eligibility criteria using variables defined with expert-abstracted and structured data (i.e., *abstracted cohort*) and subsequently those who met the cohort selection criteria using ML-extracted and structured data (i.e., *ML-extracted cohort*). We then performed analyses related to each archetype using the *abstracted cohort* and the *ML-extracted cohort*. Results and conclusions based upon these results were compared between data curation approaches.

### 2.1. Data Source

The data used to generate the results of this study were obtained from Flatiron Health’s US-nationwide EHR-derived database, which includes longitudinal de-identified data from ~280 cancer practices (approximately 800 distinct sites of care) curated via technology-enabled abstraction [5,15]. The distribution of patients across community and academic practices largely reflects patterns of care in the US, where most patients are treated in community clinics, but this can vary for each disease. Mortality information is captured via a composite variable that uses multiple data sources (structured and unstructured EHR-derived content, commercial sources, Social Security Death Index) and is benchmarked against the National Death Index data as the gold standard [16]. We obtained the key analysis variables from both structured and unstructured (e.g., physician notes, pathology reports, discharge summaries) data sources in the patient’s EHR (Table 1). A data cutoff date of 30 November 2022 was used, meaning that all information recorded into the EHR through 31 October 2022 would be included. Unstructured data were then curated by both expert clinical abstractors and ML models (Figure 1).

#### 2.1.1. Expert Abstraction

All manual abstraction of unstructured information is carried out by trained abstractors (i.e., clinical oncology nurses or tumor registrars). Clinically relevant details are abstracted from relevant forms of clinical documentation available in the EHR, including clinic visit notes, radiology reports, pathology reports, etc. Abstractors are trained to identify and extract relevant information by following policies and procedures that are tested and optimized for reliability and reproducibility through iterative processes, and oversight is provided by oncology clinicians. The database undergoes continuous audit procedures to monitor abstractor performance while proprietary technology links each curated data variable to its source documentation within the EHR, enabling a subsequent review when necessary. Further, these data undergo quality assurance/quality control procedures to ensure data conformance, plausibility, and consistency. At the individual patient level, this approach provides a recent and robust longitudinal view into the clinical course, capturing new clinical information as it is documented within the EHR.

#### 2.1.2. Machine Learning Extraction

A multi-disciplinary ML team (including oncology clinicians, engineers, quantitative scientists, and other experts) developed a set of nine distinct models for key analysis variables (Table 1) that would not otherwise be available in structured EHR or claims data. Each of the 18 variables has been extracted through NLP of clinical notes, followed by an advanced ML or deep learning model, including LSTM and XGBoost, after undergoing a rigorous development, validation, and testing process that aligns with the data and the model’s objectives. Model details, such as how they were developed, have been previously described [17]. Briefly, models are trained on the data labeled by expert abstraction to recognize, interpret, and curate free text into structured variable values in order to mimic the abstraction process. Models used between 35,710 and 211,581 expert-abstracted labels for training, validation, and testing, depending on the variable. The trained models then extracted relevant information using the same clinical documents available to the expert abstractors. In this context, NLP is used to identify sentences in relevant unstructured EHR documents (e.g., oncology visit notes, lab reports, etc.) that contain a match to one of the clinical terms or phrases. These sentences are then transformed into a mathematical representation that the model can interpret. Individual models used in this study were evaluated with the research-centric evaluation framework developed by Estevez et al. [6]. Each model’s performance was evaluated using a test set of over 3000 unique lung cancer patients.

### 2.2. Study Population

**Figure 2:**
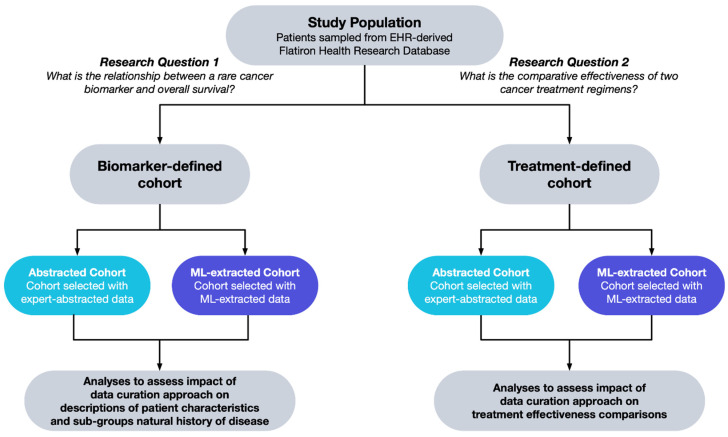
Data curation approach for replication analyses. Abbreviations: ML: machine learning.

We selected a population of patients, sampled from the study database, with the following inclusion criteria: a lung cancer ICD code (ICD-9 162.x or ICD-10 C34x or C39.9) and at least two unique-date clinic encounters documented in the EHR in the study database (reflected by records of vital signs, treatment administration, and/or laboratory tests) on or after 1 January 2011. Among this population, we applied study eligibility criteria for each research question and selected two distinct cohorts for analysis. Some of the cohort selection criteria used variables that were defined by expert-abstracted and structured data and then replicated using ML-extracted and structured data (i.e., the *abstracted* and *ML-extracted cohort*, respectively). The selection of patients for the biomarker-defined population and treatment-defined population is described in Figure 2.

#### 2.2.1. Biomarker-Defined Cohort

To answer the research question of survival by biomarker status, we selected patients diagnosed with NSCLC between 1 January 2011 and 31 October 2022 having advanced disease, defined as being either stage IIIB or higher upon diagnosis, or those who had earlier stage disease with subsequent development of recurrent or metastatic disease, and either (1) ever-positive status for a *ROS1* rearrangement after NSCLC diagnosis or (2) only negative status for a *ROS1* rearrangement in addition to a never-positive status for *ALK* (anaplastic lymphoma kinase) rearrangement, *BRAF* mutation, and *EGFR* (epidermal growth factor receptor) mutation, after NSCLC diagnosis. Patients were excluded in this cohort if they did not have a test result or only an unknown test result for the biomarker of interest, *ROS1*.

#### 2.2.2. Treatment-Defined Cohort

To answer the research question comparing the effectiveness of cancer treatment regimens, we selected a cohort of patients diagnosed with de novo stage IV non-squamous NSCLC between 1 January 2011 and 31 October 2022 who received 1L treatment with either BCP or CP. Additional eligibility criteria were applied for adequate organ function as measured by lab test results and the ECOG performance status (eligibility criteria defined in the Supplementary Materials Table S3).

### 2.3. Statistical Analysis

Statistical analyses were designed to be illustrative in demonstrating the previously defined common research archetypes. These include:

1. Defining baseline characteristics;
2. Describing natural history of disease in biomarker sub-groups;
3. Balancing populations;
4. Measuring treatment comparative effectiveness.

All analyses were first performed using the abstracted cohort and data curated by expert abstractors. Using identical methods and code, we executed the same analyses using the ML-extracted cohort and data curated by ML models. The results were then compared between data curation approaches. We used the biomarker-defined cohort to evaluate the reproducibility of archetypes 1 and 2 and the treatment-defined cohort to evaluate the reproducibility of archetypes 3 and 4.

#### 2.3.1. Defining Baseline Characteristics

We summarized select patient demographics and clinical characteristics, obtained from both structured and unstructured data sources, with descriptive statistics (i.e., median and IQR for continuous variables; n and percent for categorical variables), stratified by *ROS1* rearrangement status. Using the absolute standardized mean difference (aSMD), we compared the distribution of these characteristics within the *ROS1* rearrangement strata between the abstracted and ML-extracted cohorts. Comparisons where the aSMD was less than 0.1 were considered negligible [18]. Evaluated characteristics that were curated by ML models in the replication include: cancer histology, age at advanced diagnosis, advanced diagnosis year, group stage at NSCLC diagnosis, smoking status, treatment type received, and ever positive for: *ALK* rearrangement, *BRAF* mutation, *EGFR* mutation, or PD-L1 (programmed death-ligand 1) expression.

#### 2.3.2. Natural History of Disease in Biomarker Sub-Groups

The real-world overall survival (rwOS) was calculated as the time from advanced diagnosis date to death, using a risk set-adjusted Kaplan–Meier estimator, so that patients are only counted at risk for death once the patient has met the cohort entry criteria [19,20]. The results are stratified by the *ROS1* result (*positive* or *negative*). We compared the rwOS of patients who were *ROS1*-positive versus -negative using univariate and matched and adjusted Cox proportional hazards models to estimate the hazard ratio (HR) and 95% confidence interval (CI). The Supplementary Materials describe further details on the univariate and matched models, such as modeling and matching procedures, covariates statistically controlled for, and a robustness check to evaluate an alternative covariate selection approach for the matched model.

#### 2.3.3. Balancing Populations

To balance the baseline characteristics of patients who received different treatment regimens in the treatment-defined cohort, we fit a propensity score model [18] that included the treatment start year, age, sex, race/ethnicity, smoking status, and biomarker positivity status [21]. We assigned inverse probability weights (IPW) to weight each treatment arm.

#### 2.3.4. Comparative Effectiveness Analysis

To estimate the average treatment effect (ATE) parameter, we used the IPW weighted population from the treatment-comparison cohort. We fit a Cox proportional hazards model with a treatment group indicator (*BCP*, *CP*). We summarized the comparison of rwOS between treatment groups using the estimated HR and 95% CI.

We performed all analyses using the R programming language version 4.1.3 [22]. Institutional Review Board approval of the study protocol was obtained prior to the study’s conduct, and included a waiver for informed consent.

## 3. Results

### 3.1. Biomarker-Defined Cohort

The selection of the biomarker-defined cohort included 27,478 patients when using data curated by expert abstraction and 29,586 patients when using data curated by ML extraction. Patient attrition for this cohort when using expert-abstracted and ML-extracted data is described in Supplementary Materials Table S1.

#### 3.1.1. Defining Baseline Characteristics

There were no clinically meaningful differences in the distribution of baseline characteristics for the patients selected using expert-abstracted compared to ML-extracted variables (Table 2). The prevalence of a positive biomarker test result for *ROS1* rearrangement was 1.27% (abstracted cohort) and 1.24% (ML-extracted cohort). Among biomarker-positive patients, there were small differences (aSMD < 0.2) between the abstracted and ML-extracted cohorts in the characteristics of diagnosis year, disease stage, ECOG performance status, and treatments. There were no differences among patients who were biomarker-negative.

#### 3.1.2. Describing Natural History of Disease in Biomarker Sub-Groups

**Figure 3:**
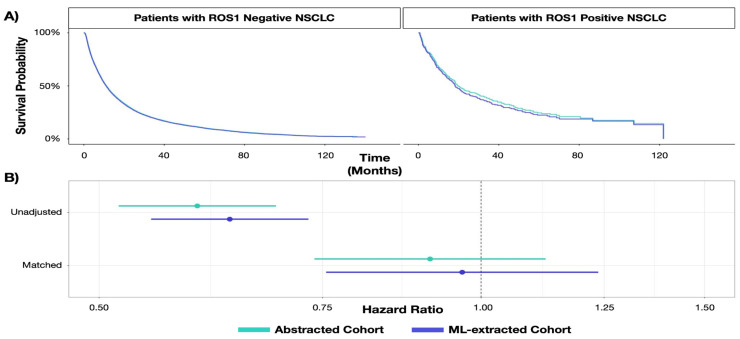
Results from replication of natural history study. Abbreviations: ML: machine learning; NSCLC: non-small cell lung cancer. (Panel (**A**)): Kaplan–Meier curves for patients with *ROS1*-positive and -negative NSCLC by data curation approach. (Panel (**B**)): Association between *ROS1* status and survival by data curation approach.

The natural history analysis of rwOS in biomarker sub-groups found the same conclusions using expert-abstracted data as with the replication using ML-extracted data. Both curation techniques found that lung cancer patients with a positive biomarker result for *ROS1* lived longer than patients with a negative result (Figure 3). From expert-abstracted data, the median rwOS was 11.28 months (95% CI: 11.02, 11.51) and 19.57 (95% CI: 17.34, 28.20) months for patients with a biomarker-negative and -positive test result, respectively. Replicating the analysis with ML-extracted data, the median rwOS was 11.05 months (95% CI: 10.82, 11.31) and 18.20 months (95% CI: 15.61, 22.79) for patients who were biomarker-negative and -positive, respectively. The relative association between biomarker result and survival did not differ between the expert-abstracted and ML-extracted data, where similar HRs and standard errors were observed (Table 3). Further, a robustness check, statistically adjusting for variables associated with *ROS1* result or survival, reached similar conclusions (Supplementary Table S2).

### 3.2. Treatment-Defined Cohort

Selection of the treatment-defined cohort included 682 patients when using data curated by expert abstraction and 701 patients when using data curated by ML extraction. The BCP treatment utilization rate was 34.60% (expert-abstracted data) and 34.52% (ML-extracted data) with other patients receiving the CP treatment regimen. Patient attrition for this cohort when using both expert-abstracted and ML-extracted data is described in Supplementary Table S3.

#### 3.2.1. Balancing Populations

**Figure 4:**
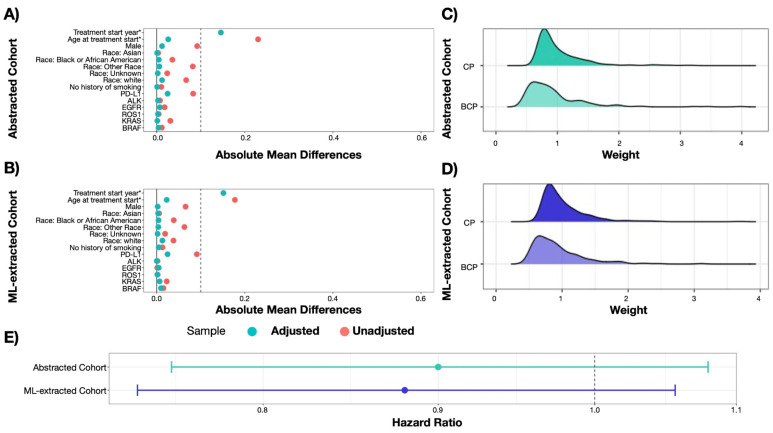
Results from replication of comparative effectiveness study. Abbreviations: 1L: first line; *ALK*: anaplastic lymphoma kinase; *EGFR*: epidermal growth factor receptor; PD-L1: programmed death-ligand 1; ML: machine learning. (Panel (**A**)): Covariate balance plot, abstracted cohort. (Panel (**B**)): Covariate balance plot, ML-extracted cohort. (Panel (**C**)): Distribution of weights stratified by treatment group, abstracted cohort. (Panel (**D**)): Distribution of weights stratified by treatment group, ML-extracted cohort. (Panel (**E**)): Effect of treatment group on survival, stratified by data curation approach.

There was no meaningful difference in the distribution of treatment propensity score weights based on the datasets having expert-abstracted compared to ML-extracted variables. After applying inverse propensity score weights to the cohorts, we observed a similar covariate balance between treatment groups in both cohorts (Figure 4). Both weighted cohorts achieved balance (absolute or standardized mean difference < 0.1) across all variables, with the exception of the treatment start year, which has a slight residual imbalance in both the abstracted and ML-extracted cohorts.

#### 3.2.2. Measuring Treatment Comparative Effectiveness

There was no meaningful difference in the result of the estimated treatment HR for rwOS based on datasets containing expert-abstracted compared to ML-extracted variables (Table 4). With expert-abstracted data, the estimated HR was 0.90 (95% CI: 0.75, 1.08), indicating slightly longer survival for patients who received BCP compared with CP. With ML-extracted data, the HR was 0.88 (95% CI: 0.74, 1.06). The HR confidence intervals were similar between expert-abstracted and ML-extracted replication, and they yielded the same statistical inference.

## 4. Discussion

This study assessed the quality and fitness-for-use of oncology EHR-derived data curated with ML-extracted variables as compared to the reference standard of expert-abstracted variables.

We replicated four common observational research archetypes for EHR-derived datasets where the analytic cohort was defined first with abstracted and second with ML-extracted data. Overall, there was no meaningful statistical or clinical difference in the results based on ML-extracted variables in reference to the benchmark of expert abstraction. In a biomarker-defined patient population, we observed similar distributions of patient characteristics. Moreover, the conclusions about an association between biomarker status and survival was consistent between data curation approaches. Likewise, in the treatment-defined cohort, the distribution of the propensity score weights was similar for expert-abstracted and ML-extracted data. The replication of a treatment comparative effectiveness analysis also produced the same results. Together, these findings demonstrate that evidence generated by analyzing ML-extracted data can lead to the same conclusions as evidence generated with abstracted data when ML models are trained on expert-labeled data and evaluated with a research-centric approach.

We showed how more efficiently curated ML-extracted data can replicate the distribution of baseline patient characteristics that were alternatively generated through labor-intensive expert abstraction from charts. This opens more opportunities to study niche populations with larger cohorts as well as adjust for potential confounders in these patient populations with confidence that the data curation approach made no difference in the study findings or conclusions.

Given the design of our study, observed differences can be attributed to variability in how a patient’s unstructured data were processed by abstractors and ML models, resulting in patients’ observed data values being discordant. Nevertheless, minor differences in the generated evidence were observed when using expert-abstracted and ML-extracted RWD. These differences did not exceed more than an 8% difference, nor did any difference amount to what would be considered statistically or clinically meaningful.

Estimates of biomarker-associated prevalence and survival obtained using ML-extracted data are consistent with previous studies [23,24,25,26,27]. Additionally, the comparative treatment effect measured in the treatment-defined cohort is consistent with similar comparisons found in the literature [28] as well as with clinical trials [28,29]. While this analysis was not powered to demonstrate a difference, the consistency of the results obtained using ML-extracted data with the results using expert-abstracted data and from external studies further highlights the fact that RWE based on ML-extracted data are reliable when obtained from an adequate and well controlled study.

A side effect of data misclassification is the distortion of type I and II error rates [30,31]. While misclassification in the ML-extracted data may exist, it did not lead to meaningfully different model standard errors. Decision makers such as payers and health technology assessment (HTA) bodies can evaluate evidence generated using ML-extracted data similarly to evidence generated with expert-abstracted data. As misclassification is a limitation of observational research, researchers who use unstructured RWD in their studies, regardless of the curation method, should continue to apply quantitative bias analyses [32] or other bias correction methods [33,34] to understand the potential impact of misclassification.

While ML extraction can generate fit-for-purpose data for observational research, there are a number of challenges that represent significant hurdles to more widespread adoption. This includes the need for generalizable, high-quality, labeled data to train ML models in order to sufficiently reflect the target population and avoid a potential bias or inadvertent exclusion of historically marginalized populations [13,35]. Low quality or noisy labels may distort the learned function between features and labels, which could lead to incorrect model predictions and/or poor model performance. Additionally, there is a need for model transparency and explainability such that model predictions can be trusted by stakeholders and therefore be more readily accepted [36]. Finally, proper model evaluation is needed to ensure that models are fair and generalizable, which requires an adequate volume of high-quality labeled test data that is not used during model training and validation [6,37].

The findings of this study should be viewed considering certain limitations. First, this study demonstrates the fit-for-purpose of an ML-extracted dataset using a limited number of results spanning two analytic cohorts. It is possible that for another study population of interest, there could be differences between the results obtained using abstracted data vs. ML-extracted data. Nevertheless, the ML-extracted variables used in this analysis were trained on high-volume, high-quality abstracted data from a large nationwide database. Additional analyses to demonstrate that ML-extracted data are fit-for-purpose and can unlock new use cases are planned for different patient populations; however, given our sample sizes and use of expert-abstracted training data, we believe we will obtain similar results. Second, this study was not implemented on a dataset independent of model development. To do so would require abstracting an additional 186,000 lung cancer patients to obtain similar cohort sizes observed in the presented analyses. While the dataset used here is not independent of model development, it is important to note that the tasks that the models were trained to perform (i.e., information extraction) are independent of the analyses performed in this study. Third, although we adjusted for potential confounders, including demographics and relevant clinical factors, there is potential bias from confounding by unmeasured covariates, missingness, treatment compliance, or measurement error. However, it is important to note that these sources of biases will similarly impact the results regardless of the data curation approach; therefore, the comparison is unlikely to be impacted. Finally, while the ML-extracted dataset used in this study draws from multiple cancer centers that are representative of patients with cancer in the US, [15] this study does not evaluate the generalizability of these models to external cancer centers that were not included in the training population. Although the models themselves are not necessarily transportable and would benefit from retraining before use in other populations, [38] this study demonstrates that the evidence generated from well-designed pharmacoepidemiological studies using a representative cohort with ML-enabled clinical depth can be generalizable.

## 5. Conclusions

In our study, we assessed the reproducibility of oncology RWE studies using ML-extracted variables in reference to the benchmark of the standard approach in retrospective research studies with manual chart review. We performed multiple example analyses representing common archetypes for the application of EHR data in oncology research and evaluated their results in support of developing reliable, fit-for-purpose RWD using ML extraction. Our results showed that ML-extracted variables can produce similar results and analytic conclusions of analyses based on expert-abstracted variables. The ability to extract high-quality data at scale through ML extraction has the potential to unlock valuable insights and advance clinical and health outcomes research, especially when quality is more broadly communicated and understood.

## Figures and Tables

**Table 1:** Study variables and EHR data source.

EHR Source Information Type	Variables Needed for Analysis	Curation Approaches
Structured data (e.g., date of birth)	Diagnoses (i.e., ICD codes) Gender Birth year Race Ethnicity Practice type ECOG performance status Medication order date Medication administration date Visit date Mortality date ^a^	Transformation, harmonization, and deduplication
Unstructured data (e.g., clinic notes, PDF lab reports, radiology images, etc.)	NSCLC diagnosis NSCLC diagnosis date Advanced NSCLC diagnosis Advanced NSCLC diagnosis date *ROS1* test result *ROS1* test date *ALK* test result *BRAF* test result *EGFR* test result *ALK* test date *BRAF* test date *EGFR* test date PD-L1 percent staining PD-L1 test result date Group stage Histology Line of therapy ^b^ Line of therapy start date ^b^	Expert abstraction OR ML-extraction

Abbreviations: *ALK*: anaplastic lymphoma kinase; *EGFR*: epidermal growth factor receptor; ECOG: Eastern Cooperative Oncology Group; ML: machine learning; NSCLC: non-small cell lung cancer; PD-L1: programmed death-ligand 1; ^a^ mortality date is a composite variable based on multiple data sources (structured and unstructured EHR data, commercial sources, and Social Security Death Index) [16]. *ML extraction was not used to define this variable*. ^b^ Line of therapy and line of therapy date are a derived variable based on both structured and unstructured data inputs.

**Table 2:** Baseline characteristics of patients, by biomarker result and data curation approach.

	ROS1-Positive			ROS1-Negative		
	Abstracted Cohort	ML-Extracted Cohort	aSMD	Abstracted Cohort	ML-Extracted Cohort	aSMD
N	349	367		27,478	29,219	
*Practice Type, n (%)*			0.02			0.01
Academic	94 (26.9%)	102 (27.8%)		3907 (14.2%)	4032 (13.8%)	
Community	255 (73.1%)	265 (72.2%)		23,571 (85.8%)	25,187 (86.2%)	
*Gender, n (%)*			0.06			0.00
Female	217 (62.2%)	218 (59.4%)		12,966 (47.2%)	13,790 (47.2%)	
Male	132 (37.8%)	149 (40.6%)		14,510 (52.8%)	15,427 (52.8%)	
*Race/ethnicity, n (%)*			0.03			0.01
Black or African American	38 (10.9%)	42 (11.4%)		2419 (8.8%)	2568 (8.8%)	
Other race ^a^	64 (18.3%)	70 (19.1%)		3637 (13.2%)	3901 (13.4%)	
Unknown	32 (9.2%)	35 (9.5%)		2794 (10.2%)	3009 (10.3%)	
White	215 (61.6%)	220 (59.9%)		18,628 (67.8%)	19,741 (67.6%)	
*Age at advanced diagnosis, median [IQR]*	65 (55, 75)	65 (54, 74)	0.02	69 (62, 76)	69 (62, 76)	0.00
*Advanced diagnosis year, n (%)*			0.16			0.04
2011	3 (0.9%)	4 (1.1%)		95 (0.3%)	105 (0.4%)	
2012	10 (2.9%)	7 (1.9%)		253 (0.9%)	272 (0.9%)	
2013	12 (3.4%)	16 (4.4%)		638 (2.3%)	676 (2.3%)	
2014	18 (5.2%)	18 (4.9%)		1147 (4.2%)	1229 (4.2%)	
2015	15 (4.3%)	14 (3.8%)		2025 (7.4%)	2091 (7.2%)	
2016	37 (10.6%)	34 (9.3%)		2647 (9.6%)	2791 (9.6%)	
2017	56 (16.0%)	52 (14.2%)		3487 (12.7%)	3719 (12.7%)	
2018	44 (12.6%)	46 (12.5%)		3726 (13.6%)	3949 (13.5%)	
2019	47 (13.5%)	44 (12.0%)		3811 (13.9%)	3997 (13.7%)	
2020	36 (10.3%)	49 (13.4%)		3713 (13.5%)	3812 (13.0%)	
2021	49 (14.0%)	53 (14.4%)		3708 (13.5%)	3917 (13.4%)	
2022	22 (6.3%)	30 (8.2%)		2228 (8.1%)	2661 (9.1%)	
*Group stage, n (%)*			0.10			0.06
Stage I	16 (4.6%)	17 (4.6%)		2331 (8.5%)	2582 (8.8%)	
Stage II	5 (1.4%)	5 (1.4%)		1387 (5.0%)	1438 (4.9%)	
Stage III	60 (17.2%)	53 (14.4%)		5514 (20.1%)	5832 (20.0%)	
Stage IV	262 (75.1%)	288 (78.5%)		17,692 (64.4%)	18,999 (65.0%)	
Group stage is not reported	6 (1.7%)	4 (1.1%)		554 (2.0%)	368 (1.3%)	
*Histology, n (%)*			0.08			0.04
Non-squamous cell carcinoma	313 (89.7%)	334 (91.0%)		20,266 (73.8%)	21,880 (74.9%)	
NSCLC histology NOS	12 (3.4%)	8 (2.2%)		1274 (4.6%)	1155 (4.0%)	
Squamous cell carcinoma	24 (6.9%)	25 (6.8%)		5938 (21.6%)	6184 (21.2%)	
*ECOG PS at advanced diagnosis, n (%)*			0.10			0.02
0	86 (24.6%)	82 (22.3%)		5549 (20.2%)	5985 (20.5%)	
1	99 (28.4%)	99 (27.0%)		7762 (28.2%)	8405 (28.8%)	
2	18 (5.2%)	17 (4.6%)		2588 (9.4%)	2788 (9.5%)	
3	5 (1.4%)	4 (1.1%)		618 (2.2%)	632 (2.2%)	
4	0 (0.0%)	0 (0.0%)		32 (0.1%)	34 (0.1%)	
Missing/not documented	141 (40.4%)	165 (45.0%)		10,929 (39.8%)	11,375 (38.9%)	
*PD-L1 status, n (%)*			0.09			0.07
Negative	57 (16.3%)	56 (15.3%)		6548 (23.8%)	6878 (23.5%)	
Positive	178 (51.0%)	189 (51.5%)		12,500 (45.5%)	13,162 (45.0%)	
Unknown	21 (6.0%)	30 (8.2%)		1117 (4.1%)	1614 (5.5%)	
Not tested	93 (26.6%)	92 (25.1%)		7313 (26.6%)	7565 (25.9%)	
*Treatment received, n (%)*			0.13			0.03
Non-oral antineoplastic	51 (14.6%)	68 (18.5%)		19,505 (71.0%)	20,662 (70.7%)	
Other oral therapy	36 (10.3%)	33 (9.0%)		2691 (9.8%)	2674 (9.2%)	
*ROS1* inhibitor	224 (64.2%)	220 (59.9%)		159 (0.6%)	139 (0.5%)	
No treatment documented	38 (10.9%)	46 (12.5%)		5123 (18.6%)	5744 (19.7%)	

Abbreviations: aSMD: absolute standardized mean difference; ECOG PS: Eastern Cooperative Oncology Group performance status; IQR: interquartile range; NSCLC: non-small cell lung cancer; PD-L1: programmed; death-ligand 1; ^a^ Patients who reported Hispanic or Latinx ethnicity, regardless of race, were included in *Other race*.

**Table 3:** Association between *ROS1* biomarker status and survival, by data curation approach.

	RWD Curation Approach	Biomarker Overall Survival HR (95% CI)	SE	*p*-Value
Unadjusted analysis	Expert-abstracted data	0.60 (0.52, 0.69)	0.073	*p* < 0.001
	ML-extracted data	0.63 (0.55, 0.73)	0.073	*p* < 0.001
Adjusted analysis	Expert-abstracted data	0.91(0.74, 1.12)	0.107	0.387
	ML-extracted data	0.97 (0.76, 1.24)	0.126	0.785

Abbreviations: CI: confidence interval; HR: hazard ratio; ML: machine learning; SE: standard error.

**Table 4:** Association between treatment regimen (CP vs. BCP) and survival, by data curation approach.

RWD Curation Approach	Treatment Effectiveness HR (95% CI)	SE	*p*-Value
Expert-abstracted data	0.90 (0.75, 1.08)	0.092	0.258
ML-extracted data	0.88 (0.74, 1.06)	0.092	0.170

Abbreviations: BCP: bevacizumab–carboplatin–paclitaxel; CI: confidence interval; CP: carboplatin–paclitaxel; HR: hazard ratio; ML: machine learning; SE: standard error.

## Data Availability

The data that support the findings of this study have been originated by Flatiron Health, Inc. Requests for data sharing by license or by permission for the specific purpose of replicating results in this manuscript can be submitted to dataaccess@flatiron.com.

## Supplementary Materials

The following supporting information can be downloaded at: https://www.mdpi.com/article/10.3390/cancers15061853/s1, Table S1: Biomarker-defined cohort attrition table; Table S2: Robustness check of the association between ROS1 status and survival, by data curation approach; Table S3: Treatment-defined cohort attrition table. References [39,40,41] have been cited in Supplementary Materials.
