# Supplementary material for: Replication of Real-World Evidence in Oncology Using Electronic Health Record Data Extracted by Machine Learning
Source: Cancers (Basel). 2023 Mar 20;15(6):1853. doi: 10.3390/cancers15061853 (PMC10046618; doi:10.3390/cancers15061853)
Supplement: Supplementary file 1 [file cancers-15-01853-s001.zip › cancers-2213590-supplementary.pdf]

SUPPLEMENTARY MATERIAL

Modeling procedures for natural history of disease in biomarker sub-groups analysis
We summarized the comparison of rwOS between *ROS1* positive and *ROS1* negative patients by estimating the hazard ratio (HR) and 95% CI with both a unadjusted and matched and adjusted Cox proportional hazard models. Further, we describe a robustness check to evaluate an alternative approach to select covariates for statistical adjustment. Each Cox proportional hazards model was fit using risk set adjustment.

<u>Unadjusted Cox proportional hazards model</u>
We fit a risk set adjusted univariable Cox proportional hazards model.

<u>Matched and adjusted Cox proportional hazards model</u>
We matched patients with *ROS1* positive NSCLC to patients with *ROS1* negative NSCLC on: age at advanced diagnosis, year of advanced diagnosis, and gender. We fit a risk set adjusted multivariable Cox proportional hazards model to the matched cohort and statistically adjusted for treatment received, PD-L1 expression, ECOG PS, group stage at NSCLC diagnosis, cancer histology, practice type, and all matching variables (i.e., age at advanced diagnosis, year of advanced diagnosis, and gender) [39]. These variables were selected prior to analysis.

We matched patients using exact matching [40]. Once matched, we assigned IPW to patients in each stratum to calculate the ATE parameter and to achieve a 1:1 ratio of patients with *ROS1* positive and *ROS1* negative NSCLC.

<u>Robustness Check</u>
An alternative approach to covariate selection is to include covariates based on their known or perceived association with the independent (i.e., *ROS1* result) or dependent variable of interest (i.e., survival) [41]. In this robustness check, we statistically controlled for variables that were significantly associated with either *ROS1* result or survival in the Abstracted and ML-extracted cohort, respectively. Evaluated covariates included treatment received, PD-L1 expression, ECOG PS, group stage at NSCLC diagnosis, cancer histology, practice type, and race/ethnicity. For both the Abstracted and ML-extract cohort, all evaluated variables were significantly ($p < 0.05$) associated with either *ROS1* result or survival and included in the Cox proportional hazards model as well as all matching variables.

Table S1. Biomarker-defined cohort attrition table.

**Inclusion-Exclusion Criterion**	**Expert Abstraction**	**ML Extraction**
Patients with a lung cancer ICD code	186,313	186,313
Diagnosed with NSCLC on or after January 1, 2011	113,648	125,774
Diagnosed with advanced NSCLC on or after January 1, 2011	77,702	79,859

At least 2 clinic visits in the real-world database occuring on different days after advanced diagnosis date	74,924	77,074
Had a ROS1 test result after initial NSCLC diagnosis	36,472	38,009
ROS1 Positive or ROS1 Negative and never for ALK, BRAF, EGFR Positive	27,827	29,586
ROS1 Status		
● *Only ROS1 Negative and never ALK, BRAF, EGFR Positive*	27,478	29,219
● *Ever ROS1 Positive*	349	367

Abbreviations: *ALK*: Anaplastic Lymphoma Kinase; *EGFR*: Epidermal Growth Factor Receptor; NSCLC: non-small cell lung cancer

Table S2. Robustness check of the association between *ROS1* status and survival, by data curation approach

RWD Curation Approach	Treatment Effectiveness HR (95% CI)	SE	P-value
Expert Abstracted Data	0.91 (0.74, 1.13)	0.107	0.389
ML-Extracted Data	0.97 (0.76, 1.24)	0.126	0.786

Abbreviations: CI: confidence interval; HR: hazard ratio; ML: machine learning; SE: standard error

Table S3. Treatment-defined cohort attrition table

Inclusion-Exclusion Criterion	Expert Abstraction	ML Extraction
Patients with a lung cancer ICD code	186,313	186,313
Diagnosed with NSCLC on or after January 1, 2011	113,648	125,774
Diagnosed with advanced NSCLC on or after January 1, 2011	77,702	79,859
At least 2 clinic visits in the real-world database occuring on different days after advanced diagnosis date	74,919	77,070
Non-squamous histology consistent with NSCLC	52,270	53,961

Received one of the two study LOTs of interest	5,576	5,576
Age 18 or older at LOT start	5,576	5,576
Adequate organ function around 1L start	3,330	3,379
ECOG PS 0-1 around 1L start	1,884	1,858
Does not have elevated blood pressure[1] around 1L start	1,634	1,601
Does not have metastasis of the central nervous system (CNS)[2] at any time before 1L start	1,633	1,598
Group stage IV at initial diagnosis	682	701
1L LOT received<br>● Bevacizumab-Carboplatin-Paclitaxel<br>● Carboplatin-Paclitaxel	 236<br>446	 242<br>459

Abbreviations: 1L: first line; ECOG PS: Eastern Cooperative Oncology Group Performance Status; LOT: line of therapy; NSCLC: non-small cell lung cancer

[1] Elevated blood pressure was defined as a diastolic blood pressure above 100 systolic blood pressure above 150.

[2] Metastasis of the CNS defined using ICD codes (ICD-9: 198.4 and ICD-10: C79.4)
